# Detection of *Campylobacter jejuni* and *Salmonella typhimurium* in chicken using PCR for virulence factor *hipO* and *inv*A genes (Saudi Arabia)

**DOI:** 10.1042/BSR20211790

**Published:** 2021-09-22

**Authors:** Khaloud M. Alarjani, Manal F. Elkhadragy, Abdulrahman H. Al-Masoud, Hany M. Yehia

**Affiliations:** 1 Department of Botany and Microbiology, College of Science, King Saud University, Riyadh 11451, Saudi Arabia; 2 Biology Department, Faculty of Science, Princess Nourah bint Abdulrahman University, Riyadh 11671, Saudi Arabia; 3 Food Science and Nutrition Department, College of Food and Agricultural Sciences, King Saud University, Riyadh 11451, Saudi Arabia; 4 Food Science and Nutrition Department, Faculty of Home Economics, Helwan University, Cairo, Egypt

**Keywords:** Campylobacter jejuni, chicken carcass, Hipo gene, invA gene, multidrug resistance, Salmonella typhimurim

## Abstract

*Campylobacter jejuni* and *Salmonella typhimurium* are the leading causes of bacterial food contamination in chicken carcasses. Contamination is particularly associated with the slaughtering process. The present study isolated *C. jejuni* and *S. typhimurim* from fifty chicken carcass samples, all of which were acquired from different companies in Riyadh, Saudi Arabia. The identification of *C. jejuni* was performed phenotypically by using a hippurate test and genetically using a polymerase chain reaction with primers for 16S rRNA and hippurate hydrolase (hipO gene). For the dentification of *S. typhimurim*, a serological Widal test was carried out using serum anti*-S. typhimurium* antibodies. Strains were genetically detected using *invA* gene primers. The positive isolates for *C. jejuni* showed a specific molecular size of 1448 bp for 16S rRNA and 1148 bp for *hipO* genes. However, the positive isolates of the *invA* gene exhibited a specific molecular size at 244 bp using polymerase chain reaction (PCR). Comparing sequencing was performed with respect to the *invA* gene and the BLAST nucleotide isolates that were identified as *Salmonella enterica* subsp. *enterica* serovar *typhimurium* strain ST45, thereby producing a similarity of 100%. The testing identified *C.* *jejuni* for hippuricase, GenBank: Z36940.1. While many isolates of *Salmonella spp.* that contained the *invA* gene were not necessarily identified as *S. typhimurim*, the limiting factor for the Widal test used anti*S. typhimurum* antibodies. The multidrug resistance (MDR) of *C. jejuni* isolates in chickens was compared with the standard *C. jejuni* strain ATCC 22931. Similarly, *S. typhimurium* isolates were compared with the standard *S. typhimurium* strain ATCC 14028.

## Introduction

Campylobacter is a gram-negative, microaerophilic genus of bacteria that is responsible for multiple gastroenteric conditions in humans [1–3]. According to the European Food Safety Authority [4], the most common type of foodborne gastroenteritis is caused by *Campylobacter jejuni* [5]. Another common bacterial infection that manifests with severity comparable to gastroenteritis is campylobacteriosis, which is an infection also caused by the Campylobacter bacterium, most commonly *C. jejuni*. While this disease is seldom life-threatening in adults, complications can arise in young people and children, even when they are healthy. In addition, older people and immunocompromised individuals may also require antibiotic therapy [6].

The common routes for the transmission of *C. jejuni* from poultry waste to humans include exposure to bird faeces during the cleaning of the coops and poor bird handling practices (such as petting and kissing) [7]. Furthermore, the bacteria can be transmitted via the handling of contaminated eggs and meat [8], during the slaughtering process, and via the water used to clean the carcass. Placing ready-to-eat foods alongside raw chickens can result in cross-contamination, as poor practices can be related to food handling or the incorrect use of kitchen utensils, such as knives and cutting boards [9].

The hippuricase test (N-benzoylglycine amidohydrolase) is considered the moat important test whereby *C. jejuni* (hippuricase positive) can be differentiated from other campylobacters (hippuricase negative). Similarly, the *hipO* gene (hippuricase) is the main basis for the differentiation between *C. jejuni* and *C. coli* [10].

Microorganisms have the ability to produce the enzyme hippurate hydrolase, which can hydrolyse substrate hippurate into glycine and benzoic acid. The formation of this enzyme does not require microorganisms to grow. Rather, the enzyme detects the microorganisms by testing the presence of glycine, one of the end products of hydrolysis. If glycine is present, a blue or deep purple colour is manifest. Hippurate reactions have been proven to be effective in the identification of *B. streptococci* [11,12]. Recently, polymerase chain reaction (PCR)-based techniques have been found to be more specific, accurate, and sensitive than phenotypic methods as a means of differentiating between *Campylobacter* species [4,13–15]. However, as *C. jejuni* or *C. coli* are the dominant *Campylobacter* isolates in human cases and poultry, it is necessary for clinical and treatment purposes to utilize simple and economical tests that can distinguish between them.

The *Salmonella* genus comprises a group of rod-shaped, gram-negative bacteria with several flagella on their cell surfaces. These bacteria belong to the family Enterobacteriaceae and are facultative anaerobes. *Salmonella* has been proven to be urease negative, unlike *Proteus sp*. Moreover, *Salmonella* can ferment glucose, although it cannot ferment lactose. Furthermore, *Salmonella* infections cause acute gastroenteritis and are the most common cause of foodborne illness outbreaks in the world. In addition, the presence of *Salmonella* bacteria in the bloodstream can induce sepsis [16]. Gastrointestinal and typhoid fever *S. typhimurium* are also common consequences of *Salmonella* infections [17]. *Salmonella* also shows zoonosis since it can jump from nonhumans to humans [17].

The pathogenicity of *Salmonella* is related to numerous virulence genes, such as the *invA, spv, fimA*, and *stn* genes, which are associated with combined chromosomal and plasmid elements. The *invA* gene is located in the genome and contains coding for one of the most important proteins on the inner membrane, which is essential for the invasion of epithelial cells [18].

The rapid detection and identification of *Salmonella* can be achieved using PCR for the *invA* gene as per the diagnostic application [19,20]. Both *S. typhimurium* and *S. enteritidis* contain the *invA* gene virulence factor and are regarded as common and clinically significant genetic markers for the serovar that causes salmonellosis worldwide [21].

Antibacterial resistance (AMR) is recognized as one of the most significant public health challenges in the contemporary global environment. AMR impacts human, animal, and environmental health. AMR is exacerbated by multidrug resistance (MDR), which poses a major challenge for clinicians since it reduces potential therapeutic responses to bacteria, such as *Campylobacter.* The clinical treatment of campylobacteriosis is currently hampered by the ineffectiveness of many existing antibiotics, with inevitable increases in related mortality rates [22–24]. The recommended treatment for campylobacteriosis is erythromycin, which is often deemed the usual drug of choice when fighting bacterial infections, including severe intestinal infections caused by *Campylobacter*. Moreover, erythromycin is the drug of choice for use in immunocompromised patients [25,26]. In addition, *Campylobacter* is sometimes treated with other antibiotics, such as tetracycline, gentamycin, and fluoroquinolone [5]. One Canadian study found that *Campylobacter spp.* detected in chicken samples and slaughterhouses were highly resistant to several other classes of antibiotics, including tetracycline and fluoroquinolone [28–30]. In many other countries, including China and Poland, the detection of antibiotic resistance in *Campylobacter spp.* has also been reported [31–33].

The resistance of *Salmonella* and other pathogens to different classes of antibiotics can be ascertained from extrachromosomal genes. Moreover, resistance genes have been related to large transferable plasmids, which may be other DNA mobile elements, such as transposons and integrons [34]. Differences in resistance to antimicrobial determinants can amass within linked clusters because classes of antimicrobials, disinfectants, and heavy metals may be linked to MDR in bacteria [35,36]. The correlations between serotypes and drug resistance can be altered. Hence, there is a need for closer international monitoring of antimicrobial resistance phenotypes in Salmonella isolates that are human or animal in origin [34].

Many antimicrobial agents have been used to prevent infections in live poultry. Moreover, these agents have also been utilized as growth promotors in the poultry industry. Therefore, these antibiotics have been ineffective in human subjects when required to treat infections [27]. Excessive use of antibiotics in animal feed has also resulted in high resistance to antibiotics [31,32].

The current research has investigated the prevalence of *Campylobacter jejuni* and *S. typhimurium* in chicken carcasses, wherein PCR was employed to assess the presence of the *hipO a*nd *invA* genes, respectively. The presence of the *invA* gene was confirmed using serological tests with control serum anti*S. typhimurium* antibodies. Finally, the multidrug resistance (MDR) of *C. jejuni* and *S. typhimurium isolates* was determined using an array of antibiotics from different classes.

## Sample collection

Fifty samples from chicken carcasses were collected from different companies (A, B, C, D and E) located in city markets in Al-Riyadh.

## Methods used to isolate *C. jejuni* and *S. typhimurium*

To activate the standard strain of *C. jejuni* ATCC 33291 and isolate *C. jejuni* from chicken carcasses, this study utilized Bolton broth (Oxoid, CM0983, Solaar House, 19 Mercers Row, Cambridge, CB5 8BZ, U.K.). The medium was autoclaved and cooled to 50°C, after which horse blood (SR 0048) and an antibiotic supplement (SR0138) were added. The medium was inoculated with the *C. jejuni* standard strain, and the sample (cotton swab rubbing on carcasses) was incubated under microaerophilic conditions using gas generating kits (Oxoid BR38) at 42°C for 3–4 days. To study the morphological characterization of Campylobacter colonies using the streaking method on Campylobacter, blood-free selective medium agar (modified CCDA-Preston, Oxoid CM0739) was supplemented with CCDA Selective Supplement (SR0155) and incubated at 42°C for 48–72 h under microaerophilic conditions. The suspected colonies of chicken carcass isolates that were akin to the standard strain colonies were preserved in glycerol at −20°C for additional testing.

Fifty-five grams of chicken was added to 225 ml of lactose broth to isolate Salmonella from the chicken carcasses. The mixture was incubated at 37°C for 24 h as a pre-enrichment step. Lactose broth culture (1 ml) was transferred to selenite cysteine broth (used as selective enrichment medium) and incubated at 37°C for 24 h. One loop of selenite cysteine broth was streaked on xylose lysine deoxycholate agar (XLD) (Oxoid code, CM0469) and incubated at 37°C for 24 h. Suspected *Salmonella* spp. appeared as colonies with black centres surrounded by white halos. Such colonies were used for further analysis.

## The hippurate test

The hippurate hydrolysis test was standardized with *C. jejuni* ATCC 33291 and *C. coli* ATCC 33559 reference strains. The manufacturer indicates that at least McFarland 4 turbidity should be used in the hippurate test. Moreover, it is important to use a high inoculum for *Campylobacter*. For standardization, the bacteria were grown on blood agar for 42 to 48 h at 42°C in a microaerobic atmosphere generated with Anoxomat (Mart®BV Microbiology Automation, Lichtenvoorde, Holland). The effect of cell suspension turbidity on the test results was determined. Thus, 14 suspensions of both strains with optical density (OD) values ranging from 0.2 to 2.8 were prepared in 0.9% NaCl. The OD of the cell suspensions was measured at 450 nm with a photometer (GENE-TRAK®, GENE-TRAK Systems, Hopkinton, MA, U.S.A.), and the turbidity was compared by eye and with a photometer in accordance with the McFarland turbidity standards. In addition, 0.5–12 was prepared from 1% barium chloride and 1% sulfuric acid. Diagnostic tablets were added, and the tubes were incubated for 4 h at +37°C. Five drops of 3.5% ninhydrin solution (Rosco Diagnostica A/S, Denmark) were added, and the results were read immediately after 10 min of reincubation. Based on the results, the optimal cell suspension turbidity for high hydrolysis testing was defined. The patient strains were retested for hippurate hydrolysis, and the bacterial suspensions were measured with a photometer and adjusted to between OD 450 0 0.8 (approximately McFarland 6) and 1.4 (approximately McFarland 10). Each strain was tested three times. All blue or purple colour reactions were read as positive, and colourless or yellow reactions were deemed to be negative.

## DNA extraction from Campylobacter and Salmonella isolates

The DNA of Campylobacter and Salmonella isolates was extracted using a QIAamp DNA Mini Kit (50) Cat No./ID: 51304 (QIAGEN GmbH-Bezirksregierung Düsseldorf, Germany) in accordance with the manufacturer’s protocols.

## Widal tests

Widal tests used anti-*S. typhimurium* and anti*-S. entertidis* control sera (SIFIN, Institüt fur Immunpräparate und Nährmedien GmbH, Berlin, Germany REF., TS 1624 and TS 1625, LOT., 880910 and 1090712, respectively) and assessed agglutination with antigens of *Salmonella*. A positive reaction is indicated by the degree of agglutination compared with the agglutination associated with the standard strains of *S. typhimurium* ATCC 14028 and *S. entertidis* ATCC 13076.

## PCR amplification and electrophoresis

Campy 16S F TTGATCCTGGCTCAGAGT Campy 16S R TTCACCCCAGTCGCTGAT, *hipO* F ACTGCAAAATTAGTGGCG, *hipO* R GAGCTTTTAGCAAACCTTCC. PCR was used to detect the invasive encoding gene (*invA* gene) in *Salmonella* isolates. A final volume of 50 μl contained *invA* gene primers F: ACAGTGCTCGTTTACGACCTGAAT and R: AGACGACTGGTACTGATCGATAA [37]. The amplification process for *C. jejuni* genes involved incubation at 94°C for 5 min, followed by 35 cycles of 94°C for 1 min, annealing at 56°C for 30 s, and elongation at 72°C for 30 s. Subsequently, there was a final extension at 72°C for 10 min. The amplification process for *S. typhimurium* was performed using the same program with the exception of an annealing temperature of 52°C. Amplified PCR products were separated on 1% agarose gels with ethidium bromide at 100 v for approximately 1 h. DNA bands were then observed under ultraviolet light. The size of the DNA of *C. jejuni* for 16S rRNA and 1148 bp for *hipO* were compared with the DNA ladder run. In addition, the bands of the *invA* gene were used for the identification of *S. typhimurium* using the same DNA ladder run. Positive isolates were then sequenced, and the GenBank BLAST program was used to ensure that the proposed primers were consistent with the target species.

## The susceptibility test for *C. jejuni*

A comparison of antimicrobial susceptibility tests for standard strains of *C. jejuni* ATCC 33291 and seven identified isolates was performed. The tested bacteria were obtained from overnight cultures inoculated from single colonies into Campylobacter blood-free selective agar (modified CCDA-Preston), (Oxoid CM0739), supplemented with CCDA selective supplement (SR 155E), which was applied to the surface of the same medium and used in the agar disk diffusion method.

Antimicrobial susceptibility testing was performed using the disk diffusion method, in accordance with the protocol of the Clinical and Laboratory Standards Institute [38]. A total of 25 different antibiotic discs (Oxoid, U.K.) were prepared and tested against *C. jejuni* strains. The diameters of the zones of inhibition (mm) were measured using the criteria recommended for *C. jejuni* [38].

## Susceptibility test for *S. typhimurium*

Salmonella isolates were employed to compare the findings emerging from the susceptibility tests for *S. typhimurium* ATCC 14028 with the other results. Following the overnight incubation of single colonies in BHI media (Oxoid, U.K.), it was possible to extract the bacterial isolates. Cultures were spread on Mueller-Hinton agar (Oxoid, U.K.), and individual plates were used for agar disk diffusion assays.

Tests were conducted on 20 different antibiotic-impregnated disks (Oxoid, U.K.), with agents belonging to seven different classes, namely, β-lactam, aminoglycoside, cyclic peptide, sulfonamide, quinolone, fluoroquinolone, and Macrobid. The diameters of the zones of inhibition (mm) for each antibiotic and isolate were recorded using criteria recommended for Enterobacteriaceae [38]. Diameter measurements were used to classify isolates as sensitive (S) or resistant (R).

## The preparation of cell extracts of *Salmonella* isolates for sodium dodecyl sulfate-polyacrylamide gel electrophoresis (SDS-PAGE)

According to Yehia and Al-Dagal (2014) [39], electrophoresis was performed at room temperature in a vertical chamber (Biometra, Germany) using a constant voltage of 100 V until the bromophenol blue tracking dye reached the bottom of the gel. A comparison was made between the whole-cell protein profiles of presumptive *Salmonella* isolates and the standard *Salmonella typhimurium* strain ATCC 14028. A high degree of similarity with standard strains was further confirmed via positive anti-*S. typhimurium* agglutination tests.

## Results and discussion

**Figure 1:**
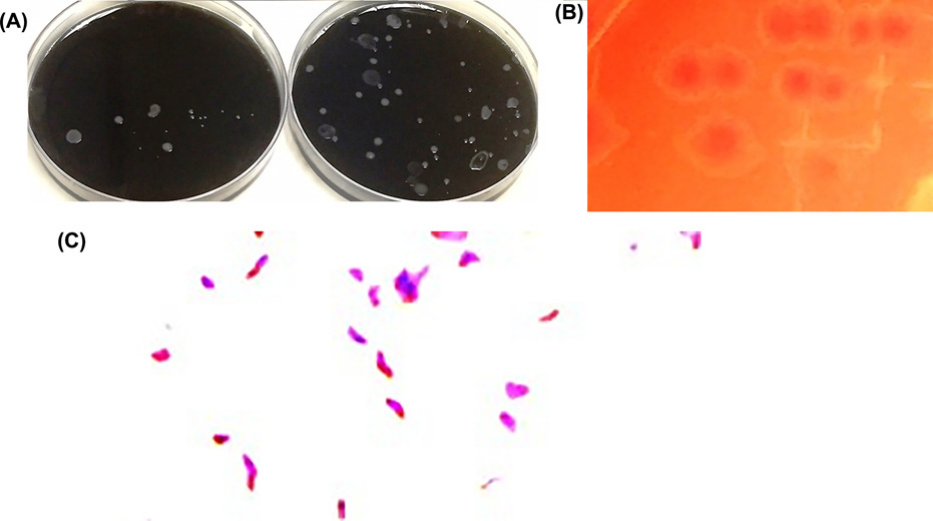
Colonies of *C. jejuni* Colonies on Campylobacter blood-free selective agar (modified CCDA-preston) (**A**) and on Bolton selective enrichment agar with agar supplements and with 25 ml of horse blood (**B**), creating a gram-negative spiral shape when stained with Gram stain (**C**).

Fifty isolates comprising 10 poultry sourced from different companies were putatively identified as *C. jejuni* based on morphological characteristics and biochemical reactions. Colonies appeared on Campylobacter blood-free selective agar (modified CCDA-preston) and on Bolton selective enrichment agar supplemented with 25 ml of horse blood, as shown in Figure 1A and B. Some of the colonies that were greyish, low, flat, finely granular, and translucent may spread and swarm, whereas others were 1–2 mm in diameter, and others exhibited small raised convex, smooth, and glistening colonies with an entire edge [40]. *Campylobacter* spp. belongs to the family Campylobacteriaceae. It is spiral or S-shaped and sometimes curved in appearance when viewed under a compound microscope, as per Figure 1C. Moreover, it has Gram-negative rods when stained with Gram stain. These rods are motile, as in the motion of a corkscrew. The motility denotes the flagella, which may comprise a single form flagellum either at one end or at both ends of the cell. Nonmotile species or species with multiple flagella have also been described [41].

**Figure 2:**
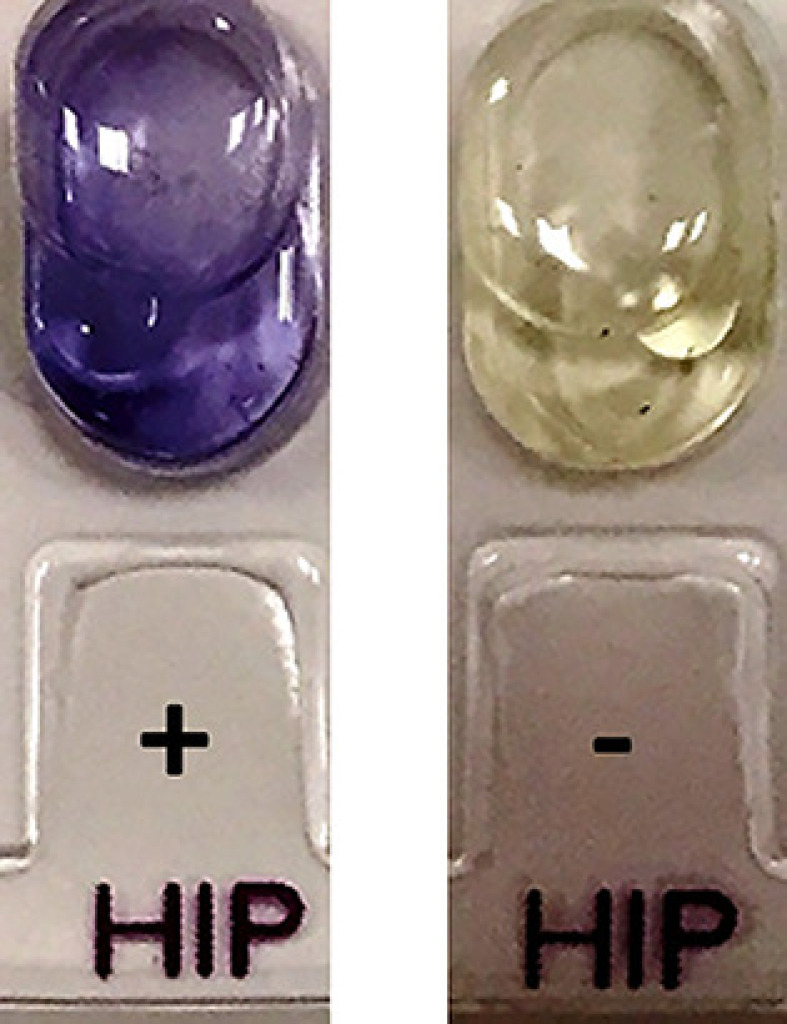
Hippurate hydrolysis test For *C. jejuni* ATCC 33291 (+) and *C. coli* ATCC 33559 (-).

To detect the ability of *Campylobacter* strains to hydrolyse hippurate, as mentioned by Hwang and Ederer (1975) [11], after a 24 h incubation period at 37°C, 50 μl of 3.5% (w/v) ninhydrin solution was added to each cupule containing a bacterial suspension and then thoroughly mixed. The samples were then viewed. All *C. jejuni* strains exhibited a dark purple color, indicative of hippurate hydrolysis. Figure 2 illustrates examples of the color changes identified in the high hydrolysis test.

**Figure 3:**
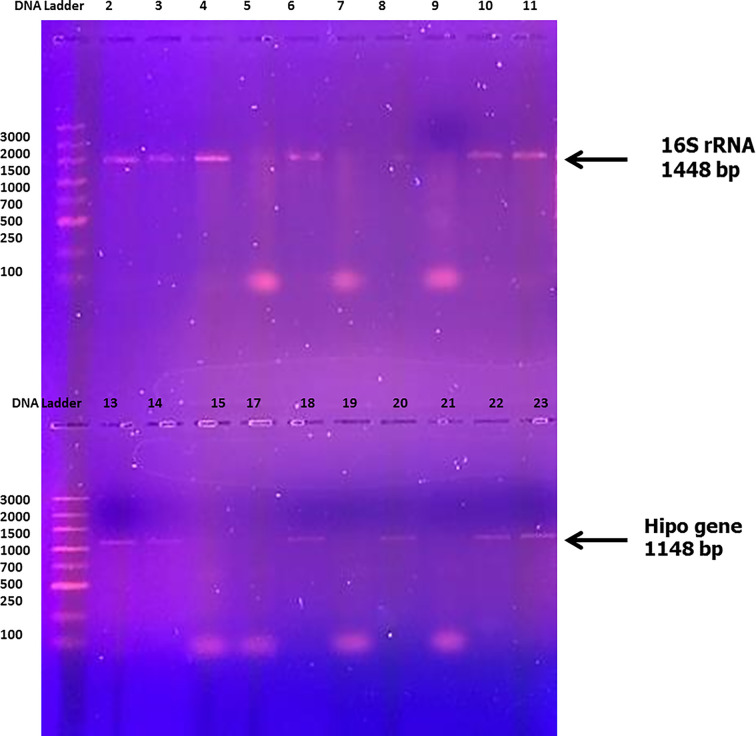
PCR amplification of **16S rRNA** and *hipO* gene primers of *C. jejuni* isolates

PCR amplification of the hippuricase (*hipO*) gene revealed that there were eight isolates that contained genes and that the other isolates failed as templates for amplification of the 1148 bp fragment (Figure 3). The isolates that were *hipO*-negative may be other *Campylobacter* species or represent *hipO*-negative *C. jejuni* [10,42]. Further analyses were required to determine their species identities. Linton et al. (1997) [42] used PCR amplification in relation to a portion of the hippuricase (*hipO*) gene isolated from humans and poultry and found that 56 of the 84 poultry isolates (67%) and 31 of the 32 human isolates (97%) contained the *hipO* gene. The outstanding 29 isolates failed as templates for the amplification of the 735 bp fragment. By using southern hybridization analyses, Linton et al. (1997) [42] found that 14 of 28 *hipO*-negative poultry isolates (50%) used a DIG-labelled 735 bp fragment of the *hip*O gene, thereby confirming that these isolates did not possess *hipO*. Thus far, the *hipO* gene has not been detected in any other species of Campylobacter; it appears to be exclusive to *C. jejuni* [43].

A number of PCR-based assays have been devised to identify specific *Campylobacter* species. However, a review of the literature failed to return any comparative analyses of the assay accuracy in classifying *Campylobacter* isolated from humans and poultry in different geographical areas. Six different published PCR assays were used to differentiate 116 *Campylobacter* isolates. Having the capacity to hydrolyse hippurate, the isolates had previously been determined to be *C. jejuni*. Our analysis verified that the majority (87 isolates) were *C. jejuni*, 28 were *C. coli* and the remaining isolate (ICPMR/6) was unidentifiable. Based upon its 16S rRNA gene sequence, this unique isolate was most homologous to the *C. upsaliensis* strain. Efforts to confirm this speculative identification of the species were unsuccessful using PCR-RFLP assays of Fermer and Engvall (1999) [44] and Jackson et al. (1996) [45]. Three PCR assays confirmed *C. jejuni* identification: PCR-RFLP [45], a putative oxidoreductase PCR assay [46], and *hipO* [42]. There was 100% agreement between the assays: 31 of the 32 human isolates and 56 of the 84 poultry were *C. jejuni*. Using the assay of Stucki et al. [42], the same number of human isolates was identified as *C. jejuni*; however, in contrast with the other assays, only 52 of the 84 poultry were identified as *C. jejuni* by this method. The poultry isolates that failed to amplify a PCR product using the assay of Stucki et al. were 98/E600/5, 98/E599/10, 99/3912/7 and 8. Possible reasons for these isolates failing to amplify a PCR product could be that there were PCR inhibitors present in the whole-cell DNA preparations or there were slight variations in the sequence of one or both primer sites. The least reliable assay was the PCR-RFLP assay devised by Fermer and Engvall (1999) [44]. In this assay, three isolates returned uncategorizable RFLP patterns, and no PCR amplicon was generated for 10 isolates. All 28 *C. coli* isolates were identified correctly by the *C. coli*-specific PCR assay [42]. However, this assay also identified one human and two poultry *C. jejuni* isolates as *C. coli*, indicating that more than one assay method should be used to verify the identification of *C. coli* species.

Totten et al. (1987) [10] recommended that the differentiation of thermophilic *Campylobacter* spp. not be centred on the single criterion of hippurate hydrolysis. They argue that while *C. jejuni* is the only *Campylobacter* species that has that capacity, some *C. jejuni* isolates are hippurate-negative. The findings from our study confirm this recommendation, as our high hydrolysis results were inconsistent in their reproducibility; additionally, weakly positive reactions were interpreted variably, and the strength of the reaction was influenced by the size of the inoculant. The uncertainty in identifying isolates is highlighted by a study conducted by Engvall et al. (2002) [47]. They recovered 174 *Campylobacter* isolates from various wild and domestic animals, of which 52 were identified incorrectly or unreliably as being either *C. jejuni, C. coli, C. lari* or *C. upsaliensis*. Together with our own, the results of Engvall et al. (2002) [47] indicate that phenotypic and biochemical assays are not reliable for determining *Campylobacter* spp., especially isolates obtained from animals.

Positive 16S rRNA and *hipO* gene amplification for *C. jejuni* ATCC 33291 (Lane 2) and isolates (Lanes 3, 4, 6, 7, 10 and 11). The DNA bands at 1448 and 1148 bp show that the marker is present.

The length of the 16S rRNA gene fragments for the eight sequenced *Campylobacter* isolates was 1448 nucleotides. Ambiguities were not detected in any of the sequences, indicating that the three 16S rRNA genes contained no sequence polymorphisms. The DNA sequence analysis of 16S rRNA genes for the eight isolates showed the highest identity to *Campylobacter sp.* strain RM12654 16S ribosomal RNA gene, GenBank: MW131451.1. While the DNA sequence of the *hipO* gene of the eight isolates shared identity with *C. jejuni* for hippuricase, GenBank: Z36940.1.

Table 1 presents data indicating that nine isolates of *Campylobacter sp.* were found in the 50 chicken carcass samples, equating to a ratio of 18%. Of the nine Campylobacter sp. isolates, five were *C. jejuni* (55.55%), as determined by PCR, which was conducted using primers for *hipO* and the 16S rRNA genes. In Indonesia, *Campylobacter* spp. in chicken meat was found to reach to 61.9%. Regarding the identification, 23 isolates (41.07%) were *C. jejuni*, 22 (39.29%) were *C. coli*, six (10.71%) were a mix between *C. jejuni* and *C. coli*, and five isolates (8.93%) were *Campylobacter* spp. The high prevalence of *C. jejuni* and *C. coli* in chicken meat in Indonesia indicates a high risk of campylobacteriosis in humans (Syarifah et al., 2020) [48].

**Table 1:** Results for *Salmonella* sp., *invA* gene, and serum anti*typhimurium* and *C. jejuni* isolate detection in chicken samples

Microorganisms		*Salmonella sp.*			Sequence identification	*Campylobacter sp.*			Sequence identification
Poultry companies	No. of samples	No. of isolates	*invA gene*	Widal test		No. of isolates	*hipO gene*	16S rRNA	
**A**	10	7	5	2	*S. enterica* subsp. *enterica* serovar *typhimurium* strain ST45 GenBank: CP050753.1	1	1	1	*C. jejuni* for hippuricase, GenBank: Z36940.1.
**B**	10	6	4	0		2	1	1	
**C**	10	9	5	2		2	1	1	
**D**	10	6	6	4		3	1	1	
**E**	10	5	3	2		1	1	1	
**Total**	50	33	23	10	10	9	5	5	5
**%**	100	66	69.69	43.47	43.47	18	55.55	55.55	55.55

*Salmonella* colonies on xylose lysine deoxylate agar (XLD) medium can be identified by their black centres and circular white halos. Microscopic examination of *Salmonella* colonies shows gram-negative rod-shaped cells. Out of 50 chicken carcasses, 33 samples (66%) were primarily identified by their growth on a specific medium, and the shape of the Salmonella sp. colony (see Table 1).

The results of Widal tests for visible agglutination between invA-positive Salmonella isolates and control sera are provided in Table 1. Anti*-Salmonella typhimurium* and anti-*S. entertidis* were applied to colonies grown on brain heart infusion agar for 24 h at 37°C. Of the 33 isolates, only 23 (69.69%) were positive for the *invA* gene, whereas 10 (43.47%) were positive in the Widal tests. The remaining 13 isolates (56.52%) were positive for the *invA* gene but negative in the Widal assays.

**Figure 4:**
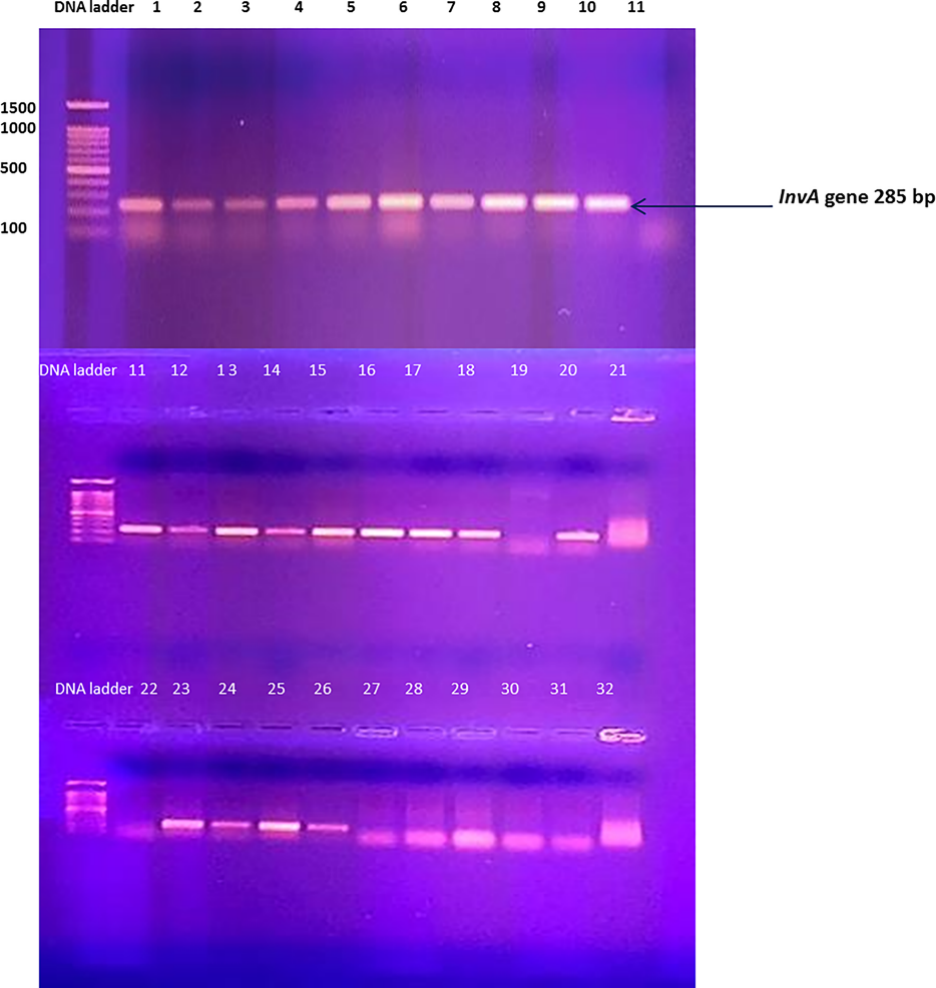
PCR amplification of *S. typhimurium invA* gene primers Positive invA gene amplification for *S. typhimurium* ATCC 14028 and *S. enetritidis* ATCC 13076 (Lane 1 and Lane 2) for 21 isolates (3-10), (11-18), (20) and (23-26). The DNA band at 244 bp shows marker presence, as visualized by gel electrophoresis using 1% agarose with an image analyser (SYNGENE) and DNA marker (1 kb ladder)

An examination of the incidence of Salmonella in poultry carcasses in different national contexts indicates that there is a huge variation in its presence. Specifically, proportions range from 3% to 66% [49,50]. The findings emerging from the current research indicate that there remains a need for substantial improvements to hygiene and safety standards in the poultry industry, in addition to the need for greater consumer awareness of this issue. Amplification results for all 33 *Salmonella* isolates revealed that only 23 isolates (69.69%) were positive for the *invA* gene, displaying a DNA marker of 244 bp (Figure 4). Submission of this 244 bp sequence to GenBank for BLAST alignment analysis indicated the presence of the genome of *S. enterica* subsp. In the *enterica* serovar *typhimurium* strain ST45 (Sequence ID: CP050753.1) for 10 isolates, the other 13 isolates were identified as *Salmonella sp.*

Amplification of the *invA* gene in the detection of the Salmonella gene is recognized as an international standard [51]. This gene encodes a protein in the inner membrane of bacteria that is responsible for invasion of host epithelial cells [52]. False-positives have been suggested to be be created by some bacterial isolates of Salmonella when an *invA* gene primer is utilized via PCR [53]. For this reason, attempts were made to assess performance in alternative published primers through the targeting of the *invA* gene.

Rahn et al. (1992) [53] used isolates from poultry such as Citrobacter spp., *E. coli* and *Serratia* sp., whereby it was possible to select *invA* gene primers with nonspecific signals. Comparable results have been reported in reactions containing genomic DNA from non-*Salmonella* isolates [51–55]. The DNA of the type of strain indicated a high specificity for *invA* PCR assays [56,57]. A study designed to evaluate the specificity of PCR assays based on an *invA* gene with a stain generated conflicting results and indicated that PCR assays based on *invA* gene amplification are not a reliable means of *Salmonella* detection.

The present study concluded that while many isolates of *Salmonella spp.* may contain the *invA* gene, not all of them may be identified as *Salmonella typhimurium*. Hence, additional tests, such as the Widal test, should be used to complete the identification of Salmonella strains.

The results in Table 2 reveal that all the identified *C. jejuni* isolated from the chicken carcass samples were resistant to different antibiotics in comparison with the *C. jejuni* ATCC 33291 standard strain. The highest level of antibiotic resistance was found in *C. jejuni* 2 A1, with a resistance ratio of 100%, followed by *C. jejuni* 2 E1, with a resistance ratio of 92.8%; *C. jejuni* 2 B, B2, with an 85.71% resistance rate; and *C. jejuni* 2 C5, C6 and D7-D9, which were 78.57% resistant. The lowest ratio was recorded for *C. jejuni* ATCC 33291, which demonstrated 57.15% resistance. Hence, there is a high change in the resistance ratio attributed to the fact that the microbes acquired resistance to multiple classes of antibiotics.

**Table 2:** The susceptibility test for *C. jejuni isolates*

Antibiotics classes	β- Lactam	Chloramphenicol	Quinolones		Oxazolidone	Aminoglycosides	Lipopeptides		Penicillin		Glycopeptide	Tetracyclines	Macrolides		%		
Isolates	AMC 30 ≥18 14-17 ≤13	C 30 ≥ 1 8 13-17 ≤12	CIP 5 ≥ 2116-20≤ 15	NA 30 ≥ 1914-18 ≤13	LZD 30 ≥ 21 – ≤ 20	K 30 ≥ 18 14-17 ≤13	N 30 ≥ 16 14-17 ≤12	CT 25 ND	TIC 75 ≥20 15-19 ≤14	AMP 25 ≥ 17-14-16 ≤ 13	VA 5 ≥ 17 15-16 ≤ 14	DO 30 ≥ 14 11-13 ≤ 10	E 15 ≥ 23 14-22 ≤13	F300 ≥ 1 7 15-16 ≤14	Resistance	Intermediate	Sensitivity
*C. jejuni* ATCC 33291	R	S	I	S	R	S	*S*	R	I	R	R	R	R	R	57.15	14.28	28.57
*C. jejuni* 2 A1	R	R	R	R	R	R	*R*	R	R	R	R	R	R	R	100	0	0
*C. jejuni* 2 B1	R	R	R	I	R	R	*R*	R	R	R	R	R	I	R	85.71	14.28	0
*C. jejuni* 2 B2	R	R	R	I	R	R	*R*	R	R	R	R	R	R	S	85.71	7.14	7.14
*C. jejuni* 2 C5	R	R	I	I	R	R	*R*	R	R	R	R	R	R	S	78.57	14.28	7.14
*C. jejuni* 2 C6	R	R	I	I	R	R	*R*	R	R	R	R	R	R	S	78.57	14.28	7.14
*C. jejuni* 2 D7	R	S	R	R	R	I	*R*	R	R	R	R	R	R	S	78.57	7.14	14.28
*C. jejuni* 2 D8	R	S	R	R	R	I	*R*	R	R	R	R	R	R	S	78.57	7.14	14.28
*C. jejuni* 2 D9	R	S	R	R	R	I	*R*	R	R	R	R	R	R	S	78.57	7.14	14.28
*C. jejuni* 2 E1	R	R	R	R	R	R	*R*	R	R	R	R	R	R	S	92.8	7.14	0
Resistance %	100	60	70	50	100	60	*90*	100	90	100	100	100	90	30	-	-	-
Intermediate %	0	0	30	40	0	30	*0*	0	10	0	0	0	10	0	-	-	-
Sensitive %	0	40	0	10	0	10	*10*	0	0	0	0	0	0	70			

Mean zones of inhibition for common antibiotics: S = Sensitive, I = Intermediate, R = Resistant, except noted above and *ND = Not Detected: treated as a common antibiotic inhibition zone. AMC = Amoxy/clav.acid (30 μg), C 30 = Chloramphenicol (30 μg), CIP 5 = Ciprofloxacin (5 μg), LZD 30 = Linezolid (30 μg), K30 = Kanamycin (30 μg), N 30 = Neomycin (30 µg), CT 25 = Colistin sulfate (25 µg), TIC 75 = Ticarcillin (75 μg), AMP 25 = Ampicillin (25 μg), VA 30 = Vancomycin (30 µg), Do 30 = Doxycyclin (30 µg), E 15 = Erythromycin (15 μg), F 300 = Nitrofurantoin (300 μg).

Many antibiotic classes, such as the β-lactam group, were associated with resistance in all identified *C. jejuni*, including *C. jejuni* ATCC 33291, where the resistance ratio was 100%. A similar situation was found with the B-lactam groups oxazolidone, lipopeptides, penicillin, glycopeptide, glycopeptide and tetracyclines, which were all associated with resistance in *C. jejuni*.

From the chicken-carcass samples that were obtained from various companies (A-E), all of the *Campylobacter* strains were determined to be MDR. Numerous other studies have also found that *C. jejuni* isolated from chickens is resistant to antibiotics; this phenomenon is attributed to antibiotics being overused and/or improperly used in animal husbandry and in humans. Therefore, the incidence of antibiotic-resistant infections is rising, and more new and resistant strains are emerging. To limit AMR and devise novel treatments for human and animal populations, new strategies are needed to characterise the resistance mechanisms used by C. *jejuni* [58]. That *C. jejuni* is developing resistance mechanisms in response to the overuse of antibiotics presents a significant serious public health risk. Research conducted by Pollett et al. (2012) [59], Szczepanska et al. (2017) [60] and Tang et al. (2017) [61] has confirmed that *C. jejuni* strains bear antibiotic resistance mechanisms. There is consensus among these studies that most of the resistance mechanisms are the product of overusing antibiotics in chicken feed and animal production, as well as in human medicine. Carcasses retrieved from slaughterhouses had the highest load of *C. jejuni*, but equipment was also contaminated with antibiotic-resistant strains of *C. coli* and C. *jejuni* [62].

Mechanisms considered to be responsible for *Campylobacter spp*. Resistance to the β-lactam class of antibiotics includes intrinsic resistance and the production of β-lactamase, meaning that the efficacy of this class of drugs is limited [63].

*C. jejuni* strains are sensitive to the aminoglycoside class of antibiotics. Drug-modified proteins are responsible for *C. jejuni* resistance to antibiotics. According to Gaudreau and Gilbert (1997) [63], *Campylobacter spp*. have numerous enzymes that confer resistance against the aminoglycoside class of antibiotics. These enzymes include 3′,9-aminoglycoside adenyltransferase, 6-aminoglycoside adenyltransferase and 3′-aminoglycoside phosphotransferase types I, III, IV and VII. Campylobacter resistance to macrolides is facilitated by ribosomal targets; the resistance mechanism can arise from point mutations in the 23S rRNA and/or ribosomal proteins L4 and L22 or enzyme-mediated methylation [64,65]. Payot et al. (2006) [66] described *C. rectus* resistance to macrolides arising from rRNA methylation. However, in *C. coli* and *C. jejuni*, macrolide resistance is attributed to point mutations in domain V of the 23S rRNA [67].

*C. jejuni* has also demonstrated resistance to ciprofloxacin and nalidixic acid, which are quinolone antibiotics. Corcoran et al. (2006) [68] described the phenomenon by which ciprofloxacin-resistant mutant *Campylobacter spp.* are an inevitable outcome following exposure to fluoroquinolone (FQ). Multiple studies have explored the rapid expansion in the number of FQ-resistant mutants occurring in chickens. Previously, *C. jejuni* was susceptible to FQ, but resistance emerged following treatment with enrofloxacin [69–72].

The *tet(O)* gene, which encodes a ribosomal protective protein (Farnell et al., 2005) [73], has been found in *Campylobacter* isolates in diverse animal species; this gene makes the bacteria resistant to tetracycline [7]. Until recently, no other *tet* resistance genes had been identified in *Campylobacter.* In response to the gene binding to an open A site on the ribosome of *Campylobacter spp*., a conformational change takes place, which dislodges the tetracycline molecule that is bound to the ribosome [74]. The CmeABC multidrug efflux pump has also been implicated in conferring *C. jejuni* resistance to tetracycline [75,64]. Another *Campylobacter spp*. resistance mechanism to tetracycline that has been proposed is a plasmid-encoded *tet(O)* gene [76], described as being transferred by plasmids in a horizontal manner between *C. jejuni* and *C. coli* in the intestinal tracts of animals and humans [77,78].

Globally, there is an increase in the resistance exhibited by both *C. jejuni* and *C. coli* against various antibiotics [79,80]. Research has explored the high level of resistance that has emerged in human and animal isolates towards aminoglycosides, fluoroquinolones and macrolides [81,82].

Poultry diseases are frequently treated by broad-spectrum antibiotics, such as macrolides and tetracyclines. These antibiotics have also been used for more than three decades to promote the growth of poultry. They increase poultry production by inhibiting the pathogenic microflora and encourage the concentrations of cadaverine and putrescine. Broad-spectrum antibiotics are also used to treat humans and animals. For example, in humans, erythromycin is used to treat campylobacteriosis [83], and tetracycline is used to treat respiratory infections [84]. Meanwhile, due to their nephrotoxic activities, aminoglycosides are not used in general therapy.

*Campylobacter spp*. Resistance genes can be horizontally transferred between *C. jejuni* strains that are present in the intestinal tracts of food animals and humans. To limit the further development and spread of MDR in Campylobacter strains, the routine practice of adding antibiotics without a veterinary prescription to poultry feed or water to either kill pathogenic bacteria or to promote growth must cease.

Antibiotic resistance and virulence profiles exacerbate the risk of foodborne infection, which is further exacerbated by a reduction in antibiotic treatment options (Sithole et al., 2021) [85].

*S. typhimurium* strains no. 19 and no. 25 demonstrated the highest level of resistance (60%) to the antibiotics tested. In addition, *S. typhimurium* strains no. 15 and no. 26 revealed 55% resistance, and *S. typhimurium* 8 and 20 were 50% resistant. The standard strains *S. typhimurium* ATCC 14028 no. 1 and no. 3 were resistant to 45% of the antibiotics (see Table 3), whereas *S. typhimurium* 5 was resistant to fewer antibiotics (40%). There were fewer changes in the resistance to antibiotics when compared to the changes in the resistance in the standard strain.

**Table 3:** The susceptibility test for *S. typhimurium* isolates

Antibiotics classes	B-lactam						Aminoglycosides				Cyclicpeptides	Sulfonamide	Quinolone		Fluoroquinolone	Oxazolidone	Macrobid		Chloramphenicol	Glycopeptide	Lincosamide	%		
Isola- tes	R I S	FOX 30 ≥ 18 15-17 ≤14	CAR 100 ND	TIC 75 ≥20 15-19 ≤14	AMC 30 ≥18 14-17 ≤13	CTX 30 ≥ 26 23-25 ≤22	S 10 ≥ 15 12-14 ≤11	CN 10 ≥ 15 13-14 ≤12	K 30 ≥ 18 14-17 ≤13	N 30 ≥ 16 14-17 ≤12	TE 30 ≥ 15 13-15 ≤ 4	RL 25 ND*	NA 30 ≥ 19 14-18 ≤13	NOR 5 ≥ 17 13-16 ≤12	OFX 5 ≥ 1 6 13-15 ≤12	LZD 30 ≥ 21 — ≤10	F 300 ≥ 1 7 15-16 ≤14	E 15 5 ≥ 23 14-22 ≤13	C 30 ≥ 1 8 13-17 ≤12	VA 30 ≥ 17 15-16 ≤ 14	MY 2 ND*	Resistance	Intermediate	Sensitive
*S. typhimurium ATCC 14028*		R	R	S	S	S	R	S	I	R	S	R	S	S	S	R	I	R	S	R	R	45	10	45
*S. typhimurium 1*		R	R	I	S	S	R	S	I	I	R	R	S	S	S	R	I	R	S	R	R	45	20	35
*S. typhimurium 3*		R	R	I	S	S	R	S	I	I	S	R	R	S	S	R	I	R	S	R	R	45	20	35
*S. typhimurium 5*		R	R	I	S	S	R	S	I	I	S	R	R	S	S	S	I	R	S	R	R	40	20	35
*S. typhimurium 8*		R	R	R	S	S	R	S	I	I	R	R	S	S	S	R	I	R	S	R	R	50	15	35
*S. typhimurium 15*		R	R	I	S	S	R	S	I	R	R	R	S	S	S	S	R	R	R	R	R	55	10	35
*S. typhimurium 19*		R	R	R	S	S	R	S	I	I	R	R	R	S	S	R	R	R	S	R	R	60	5	35
*S. typhimurium 20*		R	R	I	S	S	R	S	I	R	S	R	R	S	S	R	I	R	S	R	R	50	10	35
*S. typhimurium 25*		R	R	R	S	S	R	S	I	R	R	R	R	S	S	R	I	R	S	R	R	60	10	30
*S. typhimurium 26*		R	R	R	S	S	R	S	I	R	S	R	R	S	S	R	I	R	S	R	R	55	5	35
Resistance %		100	100	40	0	0	100	0	0	60	50	100	40	0	0	100	20	100	0	100	100	-	-	-
Intermediate %		0	0	50	0	0	0	0	100	0	0	0	60	100	0	0	80	0	0	0	0	-	-	-
Sensitive %		0	0	10	100	100	0	100	0	40	50	0	0	0	100	0	0	00	100	0	0	-	-	-

Mean zones of inhibition for common antibiotics: S = Sensitive, I = Intermediate, R = Resistant, except noted above and *ND = Not Detected: treated as a c100ommon antibiotic inhibition zone. FOX = Cefoxitin (30 μg), CAR = Carbenicillin 100 µg, TIC 75 = Ticarcillin (75 μg), AMC = Amoxy/clav.acid (30 μg), CTX = Cefotaxime 30 µg, S = Streptomycin (100 μg), CN = Gentamycin (10 µg), K30 = Kanamycin (30 μg), N = Neomycin (30 µg), TE = Tetracycline (30 μg), RL = Sulfamethoxazole (25 μg), NA = Naldioxic acid (30 μg), NOR = Norfloxacin (5 µg), OFX = Ofloxacin (5 µg), LZD 30 = Linezolid (30 μg), F 300 = Nitrofurantoin (300 μg), E 15 = Erythromycin (15 μg), C 30 = Chloramphenicol (30 μg), VA 30 = Vancomycin (30 µg). MY = Lincomycin (2 µg)

Resistance to antibiotics reached 100% for β-lactams (cefoxitin and carbencillin) and was high for sulfonamides (sulfamethoxazole trimethoprim), macrobids (erythromycin), oxazolidinone linezolid, glycopeptides (vancomycin) and lincosamides (lincomycin).

The data produced in the present study confirm that there is extensive resistance to some strains of *S. typhimurium.* The ACSSuT (AMP/CHL/STR/SMX/TET) phenotype may include resistance to amoxicillin-clavulanic acid (AUG), cefoxitin (FOX), and ceftiofur (TIO) and decreased susceptibility to AXO (MIC ≥ 4 μg/ml). TIO is a third-generation cephalosporin that was approved for use in animals in 1998 [84].

Resistance to antibiotics was 96.42% and 57.14% for aminoglycosides (streptomycin and neomycin, respectively), 78.57% for oxazolidone and linezolid and 64.28% for cyclopeptide and tetracycline. These data indicate that many *Salmonella* isolates are resistant to multiple antibiotics, which helps distribute these organisms and poses a serious problem to human public health. *S. typhimurium, S. entertidis* and all unidentified isolates remained sensitive to amoxycillin/clavulanic acid, cefotaxime, norfloxacin and ofloxacin with sensitivity ratios of 100%.

*Salmonella* strains were found to be multidrug-resistant (MDR) and have been detected in many serotypes, including *S. enterica* serotype typhimurium [83,85], in addition to *S. enterica* serotypesagona, anatum, choleraesuis, dublin, Heidelberg, Kentucky, Newport, Schwarzengrund, Senftenberg and Uganda, among others [86–88].

MDR *S. typhimurium* is no longer sensitive to ampicillin (AMP), chloramphenicol (CHL), streptomycin (STR), sulfonamides, and tetracycline (TET) and has been termed ACSSuT (AMP/CHL/STR/SMX/TET), referencing the strain carrying the blaCMY gene and others. Recently, many other strains belonging to the Enterobacteriaceae family of strains have exhibited the ACSSuT pattern and acquired MDR plasmids carrying the blaCMY gene [87].

Some strains may also display resistance to gentamicin (GEN), kanamycin (KAN), and trimethoprim-sulfamethoxazole ([SMX] COT), in addition to being resistant to disinfectants and heavy metals.

Resistance to third generation cephalosporins in *Salmonella* strains is of interest because these drugs are antibiotics of choice for treating salmonellosis in children, where fluoroquinolones are contraindicated [83].

Evidence of resistance to third generation cephalosporins in strains of *Salmonella* is significant because these antibiotics are the preferred therapeutic options in the treatment of children with salmonellosis in cases where medical reasons prohibit the use of fluoroquinolones [83]. The detection of pathogenic MDR Salmonella enterica serovars Typhimurium and Enteritidis from the caecal contents of healthy chickens in retail wet markets remains extremely alarming and has led to great public health concern [88].

**Figure 5:**
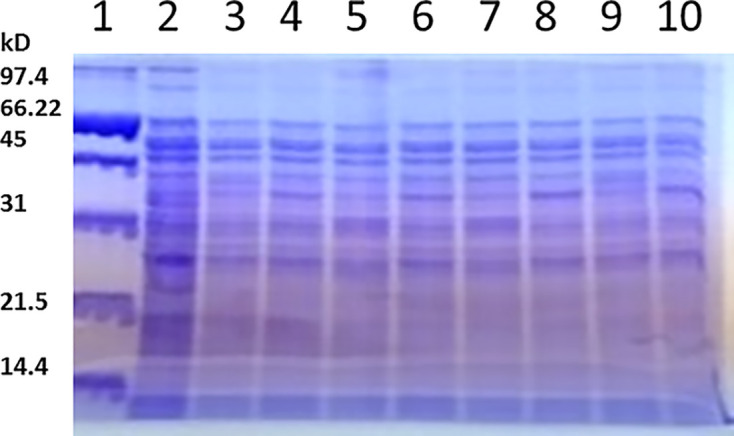
Whole protein profiles of *S. typhimurium* isolates by SDS/PAGE Lane M molecular weight standard. Lane 2 *S. typhimurium* ATCC 14028, Lanes 3–10 *S. typhimurium* total protein and a positive antisera typhimurium.

Protein profiles for ten isolates (Lanes 2–9, 12, 13) on SDS-PAGE demonstrated similar major bands compared with *S. typhimurium* ATCC 14028 (see Figure 5). The presence of the *invA* gene with positive agglutination in control sera with anti*-Salmonella typhimurium* and protein similarity for the isolates confirms the accurate identification of *S. typhimurium*. Conversely, the isolates were positive for the *invA* gene but negative in the Widal assays, which did not demonstrate the same degree of similarity in major protein bands in SDS-PAGE with the standard strains *S. typhimurium* ATCC 14028 and *S. eneteridis* ATCC 13078 (see Figure 5).

Conventional methods of Salmonella serovar identification and classification remain important for microbiological diagnosis [89]. Whole protein extracts of bacterial cells reflect the genomes of different strains and can support the identification and classification of bacteria. Comparative SDS-PAGE is also an important molecular technique used for identification at the species level [89].

Microbiological analysis related to an epidemiological investigation of outbreaks requires accurate identification and characterization of causative organisms. Many authors have used total protein extracts of Salmonella serovars on SDS-PAGE to evaluate whole cell lysates [90–94].

Our study is in agreement with Nakamura et al. (2002) [92], who reported that the total protein profiles of *S. typhimurium* ATCC 14028 and *S. enteritidis* 13076 showed major similarity in the pattern of bands on SDS-PAGE.

The finding emerging from the present study agrees with the conclusions reached by Nakamura et al. (2002) [92] that there were similarities in the SDS-PAGE band patterns in the total protein profiles for *S. typhimurium* ATCC 14028 and *S. enteritidis* 13076.

Major bands were noticed at 71.4, 67.7, 44.0, and 30.3 kDa (Nakamura et al., 2002) [92], while Ngwai et al. (2005) [94] noted that the total protein for *S. typhimurium* strains using SDS-PAGE detected 36.5 and 65 kDa proteins in all strains. Hassanain (2008) [96] observed that whole protein analysis of Salmonella by SDS-PAGE revealed that there were many bands between 11.4 and 77.5 kDa and that bands at 77.5, 55.2, 33.1, and 16.2 kDa were common. The total protein profiles of 54 Salmonella serovars, including *S. typhimurium, S. enteritidis, S. agona, S. anatum, S. virchow*, and *S. corvallis*, have also been compared using SDS-PAGE [95]. A protein band of 37.8 kDa was detected in all serovars. Furthermore, the protein profiles did not differ among the serovars. Acik et al. (2005) [93] argue that the electrophoretic banding patterns obtained using SDS-PAGE are insufficient to achieve reliable differentiation of Salmonella species.

## Conclusions

Chicken carcasses are the main disseminators of *C. jejuni* and *S. typhimurium*. Testing revealed a high prevalence of alarming microbe rates in Saudi Arabia. In addition to testing for the presence of *C. jejuni* strains, virulence factors such as the *hipO* gene and 16S rRNA are considered useful tools to assess the potential risk of chicken meat as a pathogen disseminator. The *invA* gene virulence factor was associated with the detection of *Salmonella typhimurium* and must be verified with other tests, such as Widal. However, verification by other means, such as the Widal test, is required. The present study has confirmed that the C. *jejuni* and *S. typhimurium* strains detected in chicken carcasses have levels of antimicrobial resistance, which raises the risk to human and public health.

## Data Availability

The datasets used and/or analyzed during the current study are available from the corresponding author on reasonable request.

## Abbreviations

AMP: ampicillin
CHL: chloramphenicol
GEM: gentamicin
MDR: multidrug resistance
STR: streptomycin
TET: tetracycline
XLD: xylose lysine deoxylate agar
